# Binding of adenovirus species C hexon to prothrombin and the influence of hexon on vector properties in vitro and in vivo

**DOI:** 10.1371/journal.ppat.1010859

**Published:** 2022-09-26

**Authors:** Jie Tian, Zhili Xu, Rituparna Moitra, Donna J. Palmer, Philip Ng, Andrew P. Byrnes

**Affiliations:** 1 Division of Cellular and Gene Therapies, FDA Center for Biologics Evaluation and Research, Silver Spring, Maryland, United States of America; 2 Department of Molecular and Human Genetics, Baylor College of Medicine, Houston, Texas, United States of America; UNITED STATES

## Abstract

The majority of adenovirus (Ad) vectors are based on human Ad type 5, which is a member of Ad species C. Species C also includes the closely-related types 1, 2, 6, 57 and 89. It is known that coagulation factors bind to Ad5 hexon and play a key role in the liver tropism of Ad5 vectors, but it is unclear how coagulation factors affect vectors derived from other species C Ads. We evaluated species C Ad vectors both in vitro and following intravenous injection in mice. To assess the impact of hexon differences, we constructed chimeric Ad5 vectors that contain the hexon hypervariable regions from other species C types, including vectors with hexon mutations that decreased coagulation factor binding. After intravenous injection into mice, vectors with Ad5 or Ad6 hexon had strong liver tropism, while vectors with chimeric hexon from other Ad types had weaker liver tropism due to inhibition by natural antibodies and complement. In addition, we discovered a novel ability of hexon to bind prothrombin, which is the most abundant coagulation factor in blood, and we found striking differences in the affinity of Ads for human, mouse and bovine coagulation factors. When compared to Ad5, vectors with non-Ad5 species C hexons had considerably higher affinity for both human and mouse prothrombin. Most of the vectors tested were strongly dependent on coagulation factors for liver transduction, but vectors with chimeric Ad6 hexon showed much less dependence on coagulation factors than other vectors. We found that in vitro neutralization experiments with mouse serum predicted in vivo behavior of Ad5 vectors, but in vitro experiments did not predict the in vivo behavior of vectors based on other Ad types. In sum, hexons from different human Ad species C viruses confer diverse properties on vectors, including differing abilities to target the liver.

## Author summary

Adenoviruses can be engineered to create non-replicating vectors that are useful for gene therapy or vaccination. Many Ad gene therapy vectors can specifically bind coagulation factors from the blood, and coagulation factors can influence the ability of intravenously-injected Ad vectors to deliver their genes to the liver. We show that delivery of Ad vectors to the mouse liver is more strongly dependent on coagulation factors for some Ad vectors than for others. We also demonstrate novel interactions between Ad and prothrombin, which is the most abundant coagulation factor in blood. We find that Ad vectors differ in their ability to bind to human and mouse coagulation factors, which is important because mice are a common preclinical model for studies with Ad vectors. Our studies define the interaction of Ad gene therapy vectors with coagulation factors, and the results suggest ways to design gene therapy vectors that have increased or decreased ability to target the liver after intravenous administration.

## Introduction

When non-replicating adenovirus (Ad) vectors are injected intravenously (IV), commonly-used human Ad5 vectors strongly transduce the liver, while vectors based on other Ad types sometimes have little or no hepatotropism [1–3]. Recent research on Ad5 vectors has shown that vector properties such as biodistribution and toxicity are heavily influenced by host plasma proteins that interact with Ad vectors [4]. Some of these host proteins, such as coagulation factor X (FX), bind specifically to a high-affinity binding site on hexon, which is the major Ad capsid protein [1,5]. Other host proteins such as natural antibodies and complement bind to Ad in a less specific manner, but nevertheless these other proteins can still profoundly affect vector properties *in vivo* [6–9].

Hexon has seven outward-facing hypervariable regions (HVRs) that differ markedly among Ad types [10]. These HVRs affect Ad vector tropism and neutralization by type-specific anti-Ad antibodies. The coagulation factor binding site on hexon includes several HVRs, and point mutations in these HVRs can alter the affinity of hexon for coagulation factors [11,12]. It is possible to swap HVR sequences or entire hexon sequences among different Ad types, although frequently such swaps cannot successfully be rescued [13,14]. Swapping hexon HVRs confers new properties on Ad vectors, including altered sensitivity to neutralization by type-specific antibodies [13–17], altered affinity of the Ad capsid for coagulation factors [1,12], and altered liver tropism and toxicity in mice [1,3,18–21]. Thus, mutating or exchanging the hexon HVRs of Ad vectors is a powerful tool for probing how hexon sequences determine Ad vector properties and for tuning the *in vivo* properties of Ad vectors.

Human Ad species C contains 6 types–Ad1, Ad2, Ad5, Ad6, Ad57 and Ad89 –and their hexons and other structural proteins are closely related. Ad2 and Ad5 have been shown to use the coxsackie and adenovirus receptor (CAR) as an attachment receptor and integrins as internalization receptors [22–24]. Ad5 vectors are the most frequently used Ad type for gene therapy and vaccine studies, but other Ad types are increasingly being used. For example, Ad6-based vectors are being developed as potential cancer therapeutics [18,25–27]. In mice, Ad6-based vectors have reduced liver toxicity when compared to Ad5-based vectors [26], although the situation is reversed in Syrian hamsters, where Ad6-based vectors cause more toxicity [28].

Interestingly, even though the capsid proteins of species C Ads are highly related, Ad5 vectors have notably different transduction efficiencies in mouse liver than Ad1, Ad2 and Ad6 vectors [2]. Interaction of Ad5 with CAR and integrin receptors plays only a limited role in liver transduction in mice [29], and differences in liver transduction appear to be driven primarily by hexon. For example, a chimeric Ad5 vector with the hexon of Ad6 has liver tropism that is similar to the tropism of Ad6, rather than Ad5 [18,30]. Ad57 was discovered and sequenced two decades ago [31,32], and a chimeric oncolytic vector with Ad57 hexon has been shown to have similar properties to Ad6 vectors [33]. Ad89 was identified only recently by molecular typing [34]; Ad89 has a novel penton sequence, but Ad89 hexon is nearly identical to Ad2 hexon, differing by only a few amino acids.

Many Ad types from human Ad species A, B and C have an ability to bind with high affinity to coagulation factors FX, FIX and FVII [1,5,35–37]. All coagulation factors that can bind to Ad have a Gla domain: a structural motif in which the glutamate residues are post-translationally modified by γ-carboxylation in a vitamin K-dependent enzymatic reaction [38]. Structural studies of coagulation factor binding to Ad5 show that the Gla domains of FX and FVII bind to a central cavity in trimeric hexon [1,5,11,39], and studies of mutations in hexon show that HVR5 and HVR7 are the key regions of Ad5 hexon that interact with Gla domains [5,11,12].

Other plasma proteins besides coagulation factors can also bind less specifically to Ad capsids, with profound effects on Ad vector behavior. Natural IgM antibodies are innate antibodies that can bind multivalently with low affinity and high avidity to repetitive structures such as viral capsids, and natural IgM antibodies can bind to a wide variety of viruses and bacteria, even in individuals who have had no prior exposure to those viruses and bacteria [40–42]. We have previously shown that natural IgM antibodies from mice and humans can bind Ad5, although these IgM antibodies are not neutralizing [6,8,43,44]. IgM is a strong activator of the classical complement pathway [45], and FX prevents Ad5 vectors from activating the classical complement pathway [3,7].

Systemically-delivered Ad5 vectors strongly transduce the mouse liver in a FX-dependent manner [1,5,36]. FX is required for liver transduction by Ad5 vectors because FX protects Ad5 virions and prevents their neutralization by natural antibodies and complement [7]. While the influence of coagulation factors on non-Ad5 types has received less study, distantly-related Ads are not affected by FX in the same manner as Ad5 [3]. However, among species C Ads, it is unclear whether these closely-related Ad types are affected by coagulation factors in the same manner as Ad5. Clarifying this issue is important for understanding the role of coagulation factors in Ad vector tropism and for developing vectors for clinical use.

In the current work, we used wild-type and hexon-chimeric vectors to study the interactions of coagulation factors with species C Ads, and to define the impact of hexon chimerism on Ad vector liver tropism after IV injection of mice.

## Results

### Liver transduction by helper-dependent species C Ad vectors

**Fig 1:**
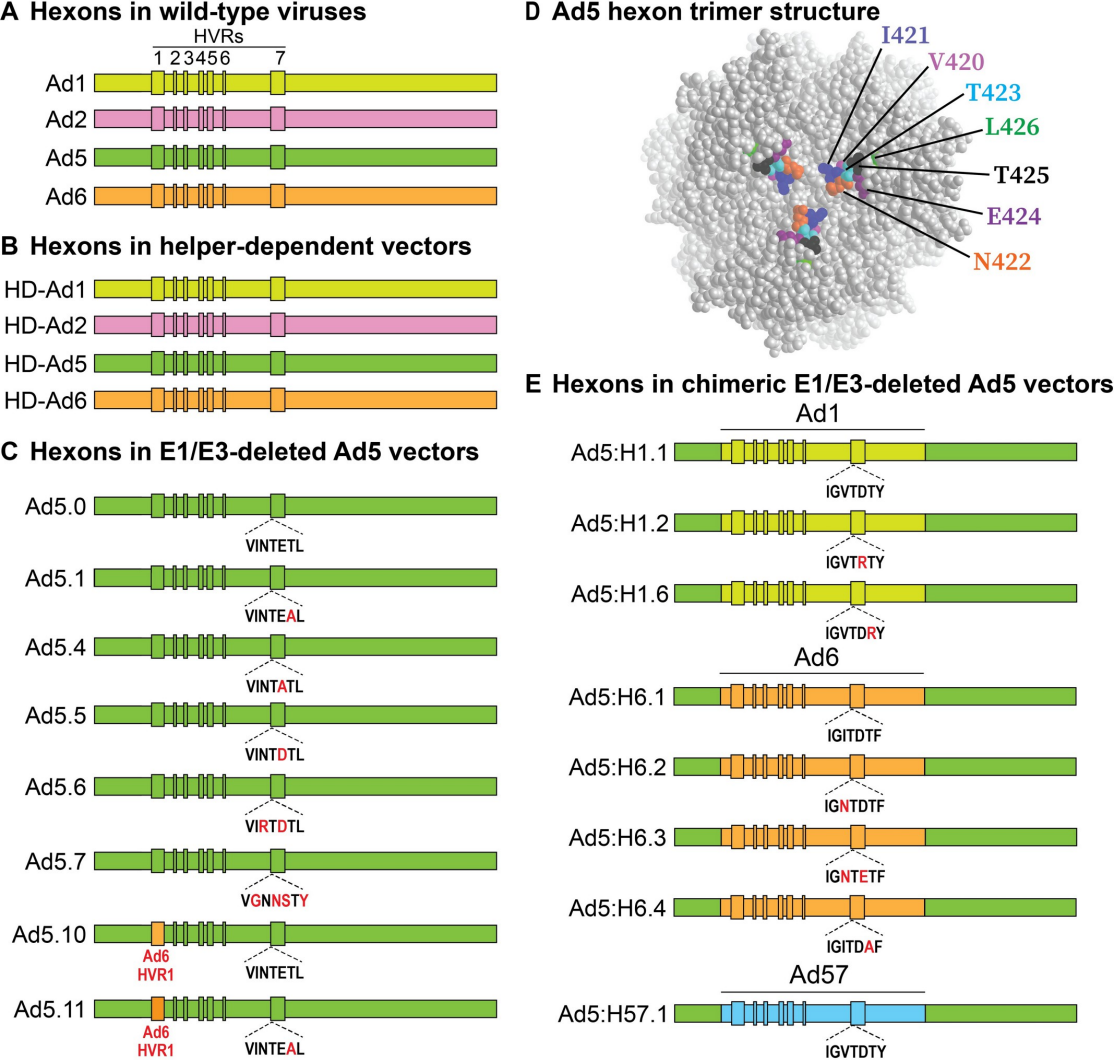
Hexons of the viruses and vectors used in this study. (A) Schematic of hexons in wild-type viruses. Each color represents a different serotype. The seven hexon hypervariable regions (HVRs) are indicated as boxes. (B) Hexons in HD vectors are identical to wild-type hexons. All HD-Ad vectors used in this study express luciferase. (C) Ad5.0 is an E1/E3-deleted Ad5 vector that expresses luciferase. We created additional vectors by site-directed mutagenesis in a short segment of HVR7 that is known to be important for Gla domain binding. Altered residues in HVR7 are shown in red. The mutations in Ad5.1 and Ad5.4 were first described by Doronin *et al*. (2012) [11], and the mutations in Ad5.7 were first described by Alba *et al*. (2009) [12]. For Ad5.10 and Ad5.11, the HVR1 region of Ad5 hexon was replaced with the HVR1 region of Ad6 hexon. (D) View of the outward-facing surface of the Ad5 hexon trimer (Protein Data Bank ID: 3TG7). The cavity in the center of the trimer contains the Gla domain binding site, and residues on the walls of this cavity affect Gla domain binding [1,5,11,12]. (E) Hexon-chimeric E1/E3-deleted Ad5 vectors that express luciferase. We replaced a section of the Ad5 hexon (residues 112–593) with the corresponding section from Ad1, Ad6 or Ad57 hexon. Remaining Ad5 hexon sequences are shown in green and are highly conserved among species C hexons (100% identity in residues 1–111, and 99.4% identity in residues 594–952). Altered residues in HVR7 are shown in red.

**Fig 2:**
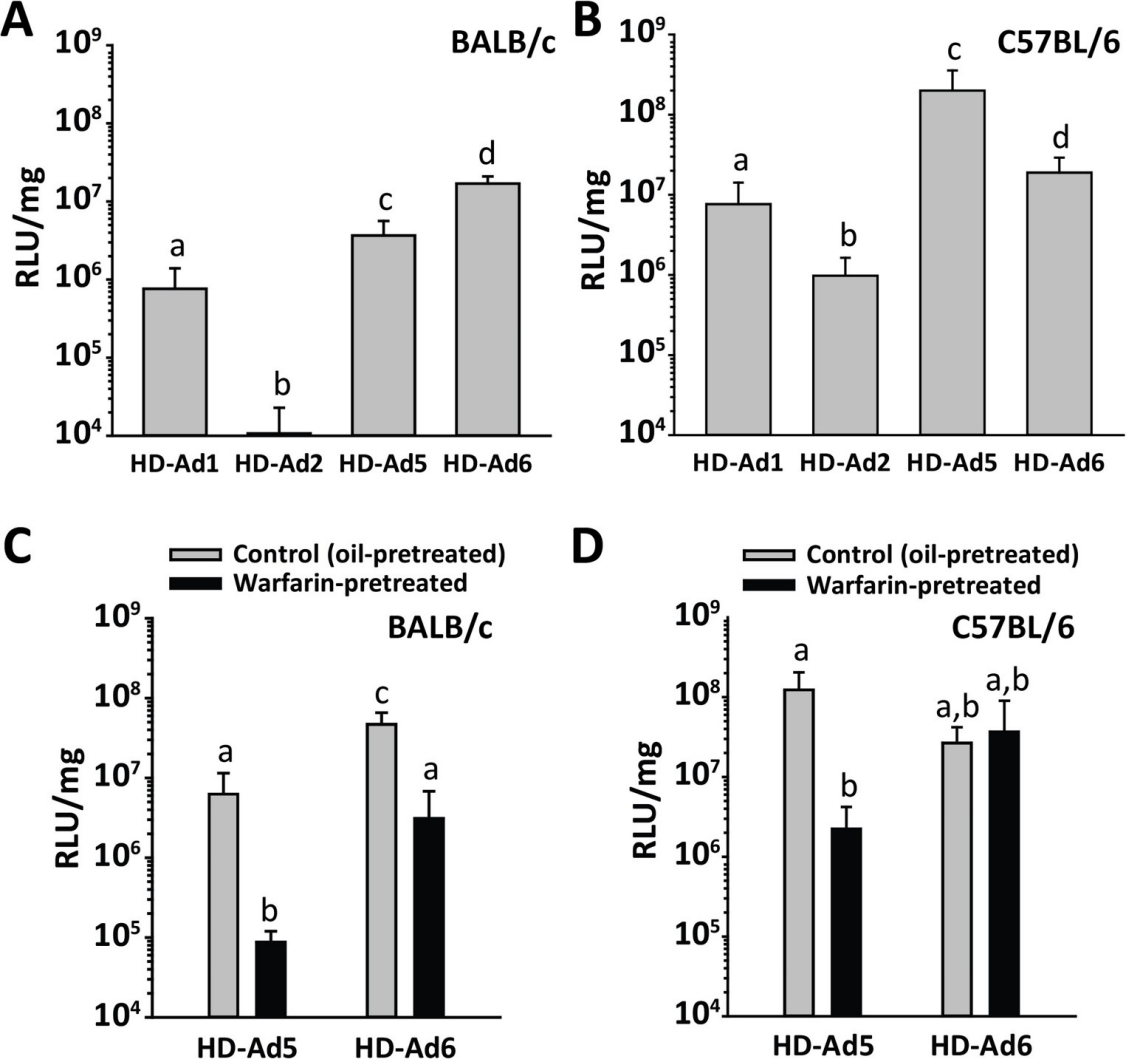
Effect of mouse strain and coagulation factors on liver transduction by species C Ad vectors. Mice were injected IV with luciferase-expressing HD-Ad vectors at a dose of 4x10^11^ vp/kg. Mice were euthanized 48 h later, and liver luciferase activity was measured. Liver luciferase activity is shown in (A) BALB/c mice and (B) C57BL/6 mice. (C) BALB/c mice or (D) C57BL/6 mice were pre-treated with warfarin to deplete coagulation factors, then injected with HD-Ad5 or HD-Ad6 vectors and evaluated for liver luciferase activity. Control mice were pre-treated with vehicle (oil) before injection with vectors. *n* = 4–7 mice per group, mean ± SD. Statistical comparisons were performed using analysis of variance (ANOVA) and the Holm-Sidak post hoc test. Groups that do not share the same letter are significantly different from each other (*P* ≤ 0.05).

IV injection of mice with Ad5 vectors leads to liver transduction, and we have previously shown that the amount of liver transduction differs among mouse strains, with liver transduction by Ad5 being substantially lower in BALB/c mice than in C57BL/6 mice due to higher concentrations of natural antibodies in BALB/c mice [8]. In BALB/c mice, interestingly, vectors with Ad6 hexon show much higher liver transduction than Ad5 vectors [2,18]. To compare liver transduction among species C vectors in these two mouse strains, we injected mice IV with helper-dependent (HD) Ad1, Ad2, Ad5 or Ad6 vectors that express luciferase. These non-replicating helper-dependent vectors contain the same capsid proteins as the corresponding wild-type Ad (Fig 1A and 1B). Helper-dependent vectors based on Ad57 or Ad89 were not available. Of the four vectors, HD-Ad6 showed the highest liver expression of luciferase in BALB/c mice (Fig 2A), in agreement with previous data from Weaver *et al* [2]. When we evaluated C57BL/6 mice, however, HD-Ad5 showed the highest liver expression of the four vectors (Fig 2B). HD-Ad2 vector had the lowest liver luciferase expression in both strains of mice, and liver transduction by HD-Ad2 vector was especially poor in BALB/c mice. Notably, the HD-Ad6 vector had similar liver luciferase expression in both mouse strains, but liver transduction by HD-Ad1, HD-Ad2 and HD-Ad5 vectors was much lower in BALB/c mice than in C57BL/6 mice (Fig 2A and 2B).

Ad5 vectors are strongly dependent on FX for liver transduction, and depletion of coagulation factors by pretreating mice with warfarin greatly reduces liver transduction by Ad5 vectors [35]. To examine whether differences between Ad5 and Ad6 vectors could be due to differing dependency on coagulation factors, we pretreated BALB/c and C57BL/6 mice with warfarin and compared liver luciferase expression after IV injection of HD-Ad5 or HD-Ad6 vectors. We found that warfarin pretreatment inhibited HD-Ad5 liver expression by over 50-fold in both strains of mice (Fig 2C and 2D). However, HD-Ad6 liver expression was unaffected by warfarin pretreatment in C57BL/6 mice (Fig 2D), and pretreating BALB/c mice with warfarin inhibited liver expression by HD-Ad6 by only 15-fold, as compared to a 72-fold inhibitory effect of warfarin pretreatment on liver expression by HD-Ad5 in BALB/c mice (Fig 2C). These results indicate that Ad6 vectors are less dependent on coagulation factors for liver transduction, compared to Ad5 vectors.

**Fig 3:**
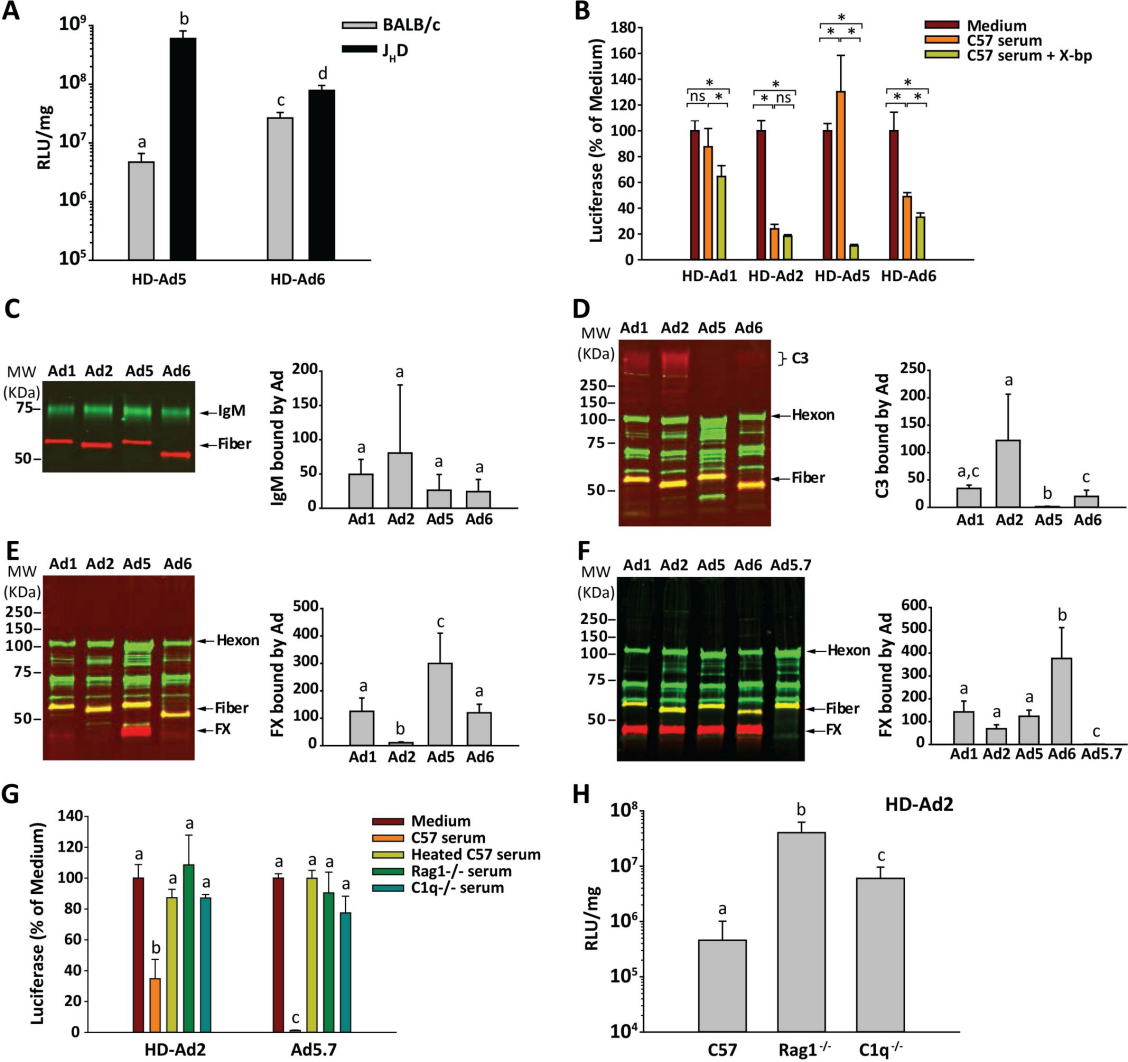
Impact of antibodies and complement on species C Ad vectors. (A) Impact of antibodies on liver luciferase activity after IV injection of HD-Ad5 and HD-Ad6 vectors. Liver luciferase activity was compared in BALB/c mice and antibody-deficient J_H_D mice on the BALB/c background. *n* = 5 mice per group. (B) Neutralization of HD-Ad vectors by mouse serum. Vectors were mixed with either medium alone or C57BL/6 mouse serum, with or without the FX-binding protein X-bp. Virus-serum mixtures were diluted and added to 293 cells, and luciferase activity was assessed one day later. *n* = 6 per group. * *P* ≤ 0.05 (ANOVA and Holm-Sidak post-hoc test). (C, D, E) Binding of mouse serum proteins to Ad virions. Wild-type Ad 1, 2, 5 and 6 were incubated for 30 min at 37°C with mouse serum, and virions were separated from non-bound serum components by density gradient ultracentrifugation. Virions and their bound proteins were assessed by quantitative western blot for binding of (C) IgM, (D) C3 or (E) FX. One representative western blot is shown, and graphs show the mean and SD of 5 samples per group. (F) Purified mouse FX was mixed with Ad virions, and bound FX was analyzed by western blot. Ad5.7 is a mutant Ad5 vector that is unable to bind FX, used here as a negative control. *n* = 3 per group. (G) The role of antibodies and complement in neutralization of HD-Ad2 vector by mouse serum. HD-Ad2 or Ad5.7 (non-FX-binding Ad5 vector) was mixed with mouse serum, and the ability of vector to transduce 293 cells was assessed by measuring luciferase activity. In addition to serum from C57BL/6 mice, we used heat-treated (complement-inactivated) serum, serum from antibody-deficient mice (*Rag1*^-/-^), and serum from mice lacking the classical complement pathway (*C1q*^-/-^). *n* = 3 per group. (H) Liver transduction by HD-Ad2 vector in C57BL/6, *Rag1*^-/-^ or *C1q*^-/-^ mice. *n* = 4 or 5 mice per group. Statistical comparison was performed by ANOVA and Holm-Sidak post-hoc tests, and groups that do not share the same letter are significantly different from each other (*P* ≤ 0.05).

Natural IgM antibodies suppress liver transduction by Ad5 vectors, and BALB/c mice have considerably higher circulating concentrations of IgM than C57BL/6 mice [8]. To determine whether liver transduction by Ad6 vectors is suppressed by natural antibodies to a similar extent as for Ad5 vectors, we examined the ability of HD-Ad5 and HD-Ad6 vectors to transduce the livers of J_H_D mice, which are antibody-deficient mice on the BALB/c background [46]. In agreement with previous data on liver expression from Ad5 vectors [7], the HD-Ad5 vector gave considerably higher (128-fold) luciferase expression in the livers of J_H_D mice than in BALB/c mice (Fig 3A). In contrast, the HD-Ad6 vector had only 3-fold higher expression in J_H_D mice than in BALB/c mice. Considering the behavior of Ad5 and Ad6 vectors in mice with high levels of antibodies (BALB/c), moderate antibodies (C57BL/6) and no antibodies (J_H_D) (Figs 2A and 2B and 3A), these data confirm our previous results that Ad5-mediated liver transduction is strongly inhibited by natural antibodies [8], and add the finding that liver transduction by Ad6 vectors is only modestly inhibited by natural antibodies. Of note, in strains where natural antibodies were either limited or absent (C57BL/6 mice in Fig 2B and J_H_D mice in Fig 3A), the Ad5 vector gave much higher liver expression than the Ad6 vector. Altogether, these results indicate that Ad5 vectors have inherently higher ability to transduce liver than Ad6 vectors in the absence of natural antibodies, but Ad5 vectors are much more sensitive to inhibition by natural antibodies than Ad6 vectors.

We next examined the interactions of the four vectors with serum and serum proteins *in vitro*, because FX is known to protect Ad5 from neutralization by natural antibodies and complement in mouse serum [7]. As expected from previous results, mixing HD-Ad5 vector with mouse serum only led to neutralization when FX was blocked with the FX-binding protein X-bp (Fig 3B). The other species C vectors showed different behavior, with HD-Ad2 being strongly neutralized by mouse serum even without X-bp. Blocking FX with X-bp only slightly increased the ability of mouse serum to neutralize HD-Ad1 and HD-Ad6. These results indicate that Ad2 is extremely susceptible to neutralization by mouse serum, and only Ad5 is strongly protected by FX in mouse serum. Ad1 and Ad6 vectors show intermediate resistance to neutralization by mouse serum.

To directly examine the ability of FX and other proteins in mouse serum to bind to virions, we incubated wild-type Ad1, Ad2, Ad5 and Ad6 viruses with mouse serum and examined binding of IgM, complement C3 and FX (Fig 3C–3E). In this assay, viruses were incubated with mouse serum for 30 min at 37°C, which leads to binding of mouse proteins and, for some viruses, activation of C3 and covalent attachment of C3 to virions [3,7]. After incubation in serum, viruses were separated from non-bound serum proteins by centrifugation and analyzed by quantitative western blot.

We found that there were no significant differences in the amount of IgM bound to the four wild-type species C Ads (Fig 3C). We next evaluated C3, because we have previously shown that FX protects Ad5 against attachment of C3 to virions [7]. When the four viruses were incubated with mouse serum, there was minimal binding of C3 to Ad5, as expected (Fig 3D). However, C3 bound strongly to Ad2, and C3 bound in moderate amounts to Ad1 and Ad6 (Fig 3D). The pattern of C3 binding was inversely mirrored by the pattern of FX binding: incubation of Ad5 with serum led to strong FX binding, binding of FX to Ad2 was very weak, and moderate amounts of FX were bound to Ad1 and Ad6 (Fig 3E). The weak binding of FX to Ad2 was surprising, as it has previously been shown that mouse FX binds to both Ad2 and Ad5 with nanomolar affinity [5]. When we mixed Ad viruses with purified mouse FX (instead of mouse serum) and used the same western-based assay, we found that mouse FX bound well to all four viruses (Fig 3F), although there was significantly more FX bound to Ad6, for unknown reasons. The relative inability of FX to bind Ad2 in the milieu of mouse serum suggests that a substance in mouse serum may prevent binding of FX to Ad2.

To further understand the high sensitivity of Ad2 vector to neutralization by mouse serum and the poor liver transduction by Ad2 vector in mice, we examined the inhibitory effects of antibodies and complement on HD-Ad2 *in vitro* and *in vivo*. When HD-Ad2 vector was incubated with C57BL/6 mouse serum, the vector was strongly neutralized (Fig 3G). However, there was no neutralization of HD-Ad2 vector by heat-treated C57BL/6 mouse serum, or by serum from antibody-deficient mice (*Rag1*^-/-^), or by serum from mice deficient in the classical complement pathway (*C1q*^-/-^). This antibody-dependent and complement-dependent pattern of neutralization of HD-Ad2 was similar to the pattern of neutralization of Ad5.7, a non-FX-binding mutant Ad5 vector, although HD-Ad2 was not as completely neutralized by C57BL/6 serum as Ad5.7 was (Fig 3G). When HD-Ad2 vector was injected IV in mice, liver luciferase expression was significantly greater in antibody-deficient or C1q-deficient mice, compared to wild-type C57BL/6 mice (Fig 3H). These results indicate that Ad2 is much more sensitive than Ad5 to the inhibitory effects of natural antibodies and complement *in vitro*, and antibodies and complement strongly restrict liver transduction by Ad2 vector *in vivo*.

### Binding of FX and FII to Ad: Effects of hexon chimerism or mutation

Comparison of FX binding to Ad virions in Fig 3E and 3F suggested that a substance in mouse serum may inhibit binding of FX to non-Ad5 species C viruses. The Gla domain of FX binds to a cavity in the center of the Ad5 hexon trimer [1,5], and the Gla domains of other coagulation factors such as FVII have also been shown to bind to the same binding site [39]. The most abundant Gla-domain-containing protein is prothrombin (FII), which is present in plasma at a concentration of 100 μg/mL (1,390 nM), as compared to FX at a concentration of 10 μg/mL (170 nM). However, it has not been reported whether FII can bind to Ads. We therefore explored whether FII could bind to species C Ads and whether FII might compete with FX for binding to the same site on hexon.

**Fig 4:**
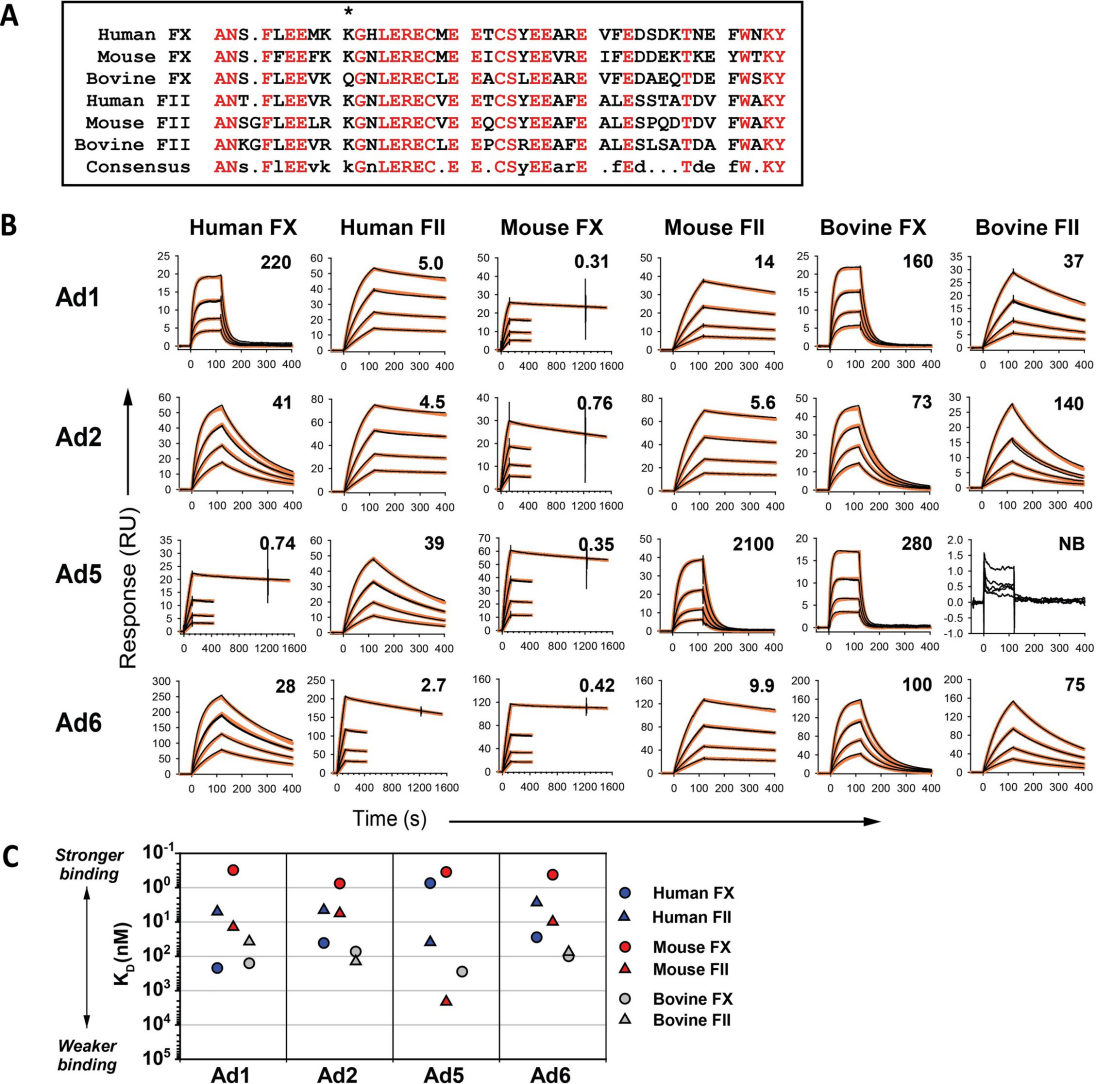
Binding of FX and FII to wild-type species C Ads. (A) Comparison of the Gla domain sequences of FX and FII. * indicates K10 of human FX, which has been proposed to interact directly with the FX binding site on Ad5 hexon [11]. (B) Wild-type adenoviruses were immobilized, and the binding of human, mouse and bovine FX and FII were evaluated using SPR. Experimental data are shown in black, and fitted curves are shown in orange. Kinetic binding affinities (K_D_) are indicated in nM on each graph, and were calculated using global fitting to a 1:1 interaction model. NB: No binding. (C) Graphical display of binding affinities from the SPR experiments in panel B.

We used surface plasmon resonance (SPR) to measure the affinities of FX and FII for binding to wild-type Ad1, Ad2, Ad5 and Ad6. Gla domains have considerable conservation among mammalian species (Fig 4A). Therefore, we compared binding of human, mouse and bovine coagulation factors to evaluate possible differences between humans and mouse models, as well as to provide data on the relationship between Gla domain sequence and affinity of binding to hexon. Interestingly, human and mouse FII bound with nanomolar affinity to Ad1, Ad2 and Ad6, but with notably reduced affinity to Ad5 (Fig 4B and 4C). Regarding affinity of FX binding, we found that mouse FX bound with sub-nanomolar affinity to all four wild-type viruses, but human FX bound with much higher affinity to Ad5 than to Ad1, Ad2 and Ad6. Bovine FX and FII bound to Ads with similar or lower affinity than the corresponding human and mouse coagulation factors.

**Fig 5:**
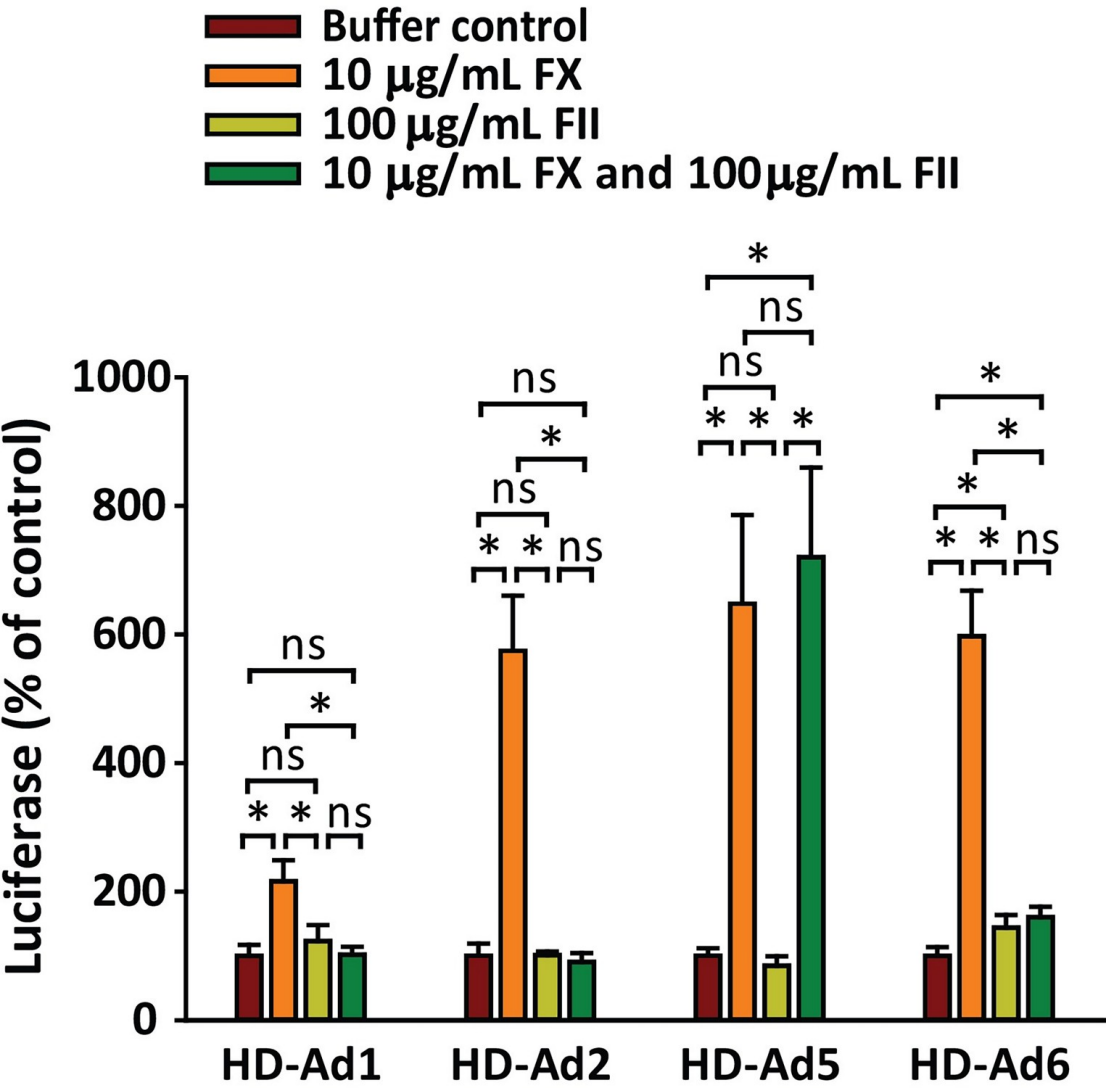
Enhancement of cellular transduction by human FX, but not human FII. SKOV3 cells were transduced with HD-Ad vectors in the presence of physiological concentrations of human FX (10 μg/mL), human FII (100 μg/mL), or both. Luciferase expression was measured 24 h later. *n* = 3 per group. Statistical differences were evaluated by ANOVA and Holm-Sidak post-hoc test (* *P* ≤ 0.05).

It has previously been shown that human FX can enhance transduction of cells by Ad5 vectors via a positively-charged region of FX that confers an ability to bind to cellular heparan sulfate [47–49]. Even though we found that Ad5 can bind to human FII (Fig 4B), it has been reported that human FII has no effect on transduction of cells by Ad5 [35]. To confirm the different transduction-enhancing effects of FX and FII and to examine the potential for competition between these two coagulation factors, we investigated how physiological concentrations of human FX and FII affect the ability of HD-Ad vectors to transduce cells. As expected, FX enhanced the transducing ability of all four vectors, although the effect on HD-Ad1 vector transduction was much weaker than for other vectors (Fig 5). In confirmation of the results of Parker *et al*. with Ad5 vectors [35], FII had no effect on transduction by HD-Ad5 vector, and similarly we found no major effect of FII on transduction by HD-Ad1, HD-Ad2 or HD-Ad6 vectors. However, when vectors were incubated together with both FX and FII, only the HD-Ad5 vector showed enhanced transduction activity. Thus, human FII competitively inhibits the ability of human FX to facilitate transduction by Ad1, Ad2 and Ad6 vectors, but not Ad5 vector.

**Fig 6:**
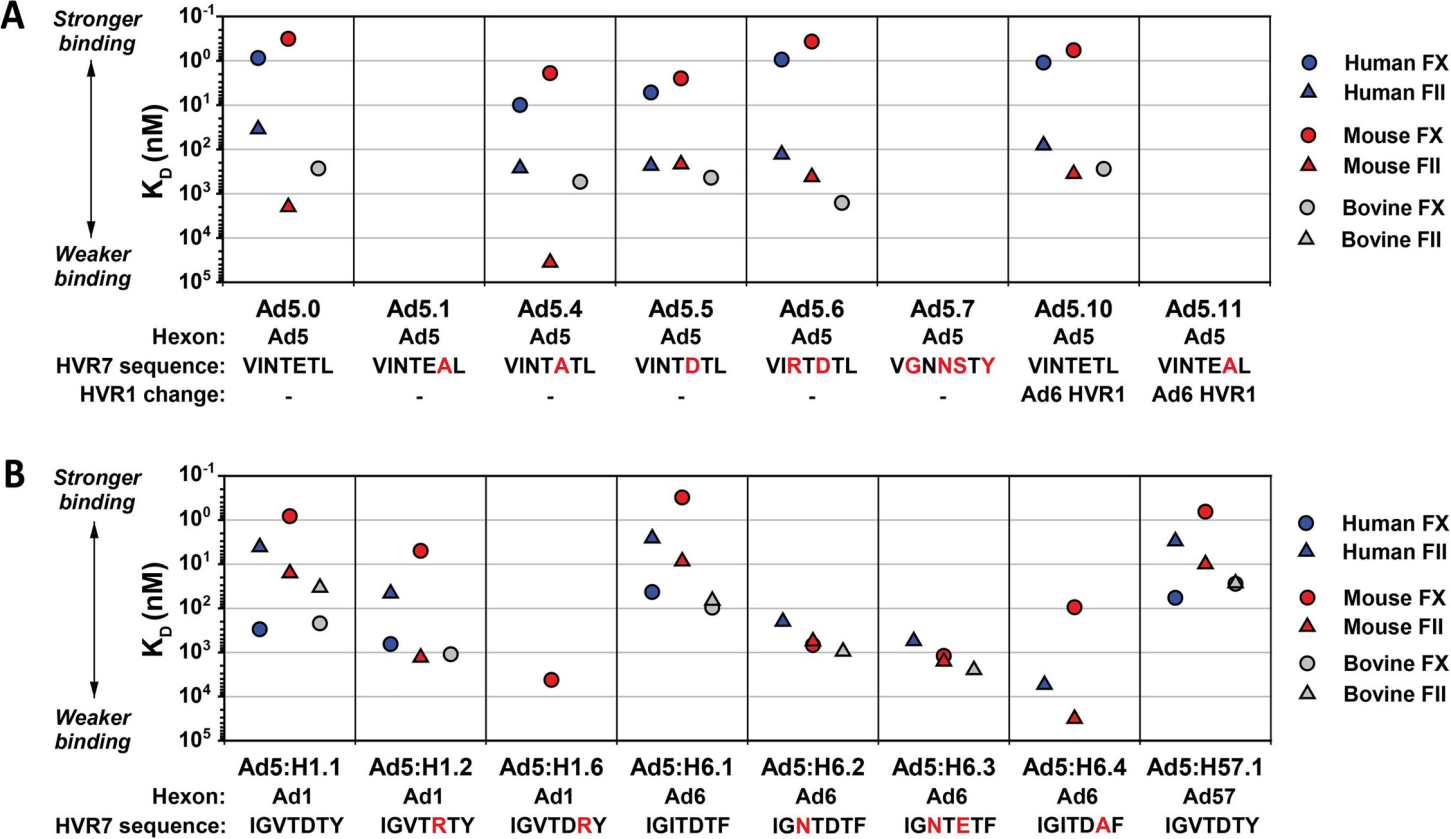
Binding of FX and FII to Ad5 vectors, hexon-chimeric Ad5 vectors, and vectors with mutations in hexon HVR7. (A) Binding affinities of FX and FII for Ad5 vectors with wild-type Ad5 capsid (Ad5.0) or vectors with various alterations in Ad5 hexon. SPR plots are presented in S1 Fig. (B) Binding affinities of FX and FII for Ad5 vectors with chimeric hexon from Ad1, Ad6 or Ad57. Mutated residues in HVR7 are indicated by red letters. The lack of a symbol signifies that binding was undetectable or the affinity could not be measured. SPR plots are presented in S2 Fig.

To examine the role of hexon and to define the FX and FII binding sites on hexon in more detail, we constructed a series of Ad5 vectors that contain chimeric hexons from Ad1, Ad6 or Ad57 (Fig 1E). We also constructed Ad5 vectors with mutated hexon HVR7, with the goal of altering the Gla domain binding site in the central cavity of the hexon trimer (Fig 1C and 1D). For vectors containing Ad5 hexon, mutations in hexon HVR7 that have previously been reported to abolish FX binding also abolished FII binding (vectors Ad5.1 and Ad5.7 in Figs 6A and S1). This result demonstrates that FX and FII bind to the same site on Ad5 hexon.

The highly-charged HVR1 of hexon is important for interaction of Ad5 with scavenger receptors, natural IgM and Kupffer cells [21,50,51]. We speculated that HVR1 of Ad6 might be responsible for some of the desirable properties of Ad6 vectors *in vivo*, and therefore we created Ad5 vectors that had the Ad5 HVR1 replaced by the Ad6 HVR1 (Fig 1C, vectors Ad5.10 and Ad5.11). It has been suggested that HVR1 might play a role in binding of FX to Ad5 hexon [52], but we found that replacing HVR1 had no major effect on FX or FII affinity (Figs 6A and S1, vectors Ad5.10 and Ad5.11, compared to Ad5.0 and Ad5.1).

When we created Ad5 vectors that had hexon sequences from Ad1 or Ad6 (vectors Ad5:H1.1 and Ad5:H6.1 in Figs 6B and S2), we found that these vectors had essentially the same affinity for coagulation factors as the corresponding wild-type virus (Fig 4B and 4C; viruses Ad1 and Ad6), demonstrating that the binding affinities of these viruses for FX and FII are completely determined by hexon. Point mutations in the HVR7 region of Ad1 or Ad6 hexon resulted in reduced binding affinities for FX and FII (Figs 6B and S2), demonstrating the critical role of HVR7 in determining the affinity of Ad1 and Ad6 for coagulation factors. We also created a chimeric Ad5 vector with the Ad57 hexon (Ad5:H57.1), and we found that the affinity of Ad57.1 for FX and FII (Figs 6B and S2) was similar to affinities seen with Ad1, Ad2 and Ad6 (Fig 4B and 4C).

Overall, Ad5 had very high affinity for human and mouse FX, while the other species C Ads had reduced affinity for human FX. In contrast, Ad5 had moderate to low affinity for human and mouse FII, while the other species C Ads had high affinity for human and mouse FII.

**Fig 7:**
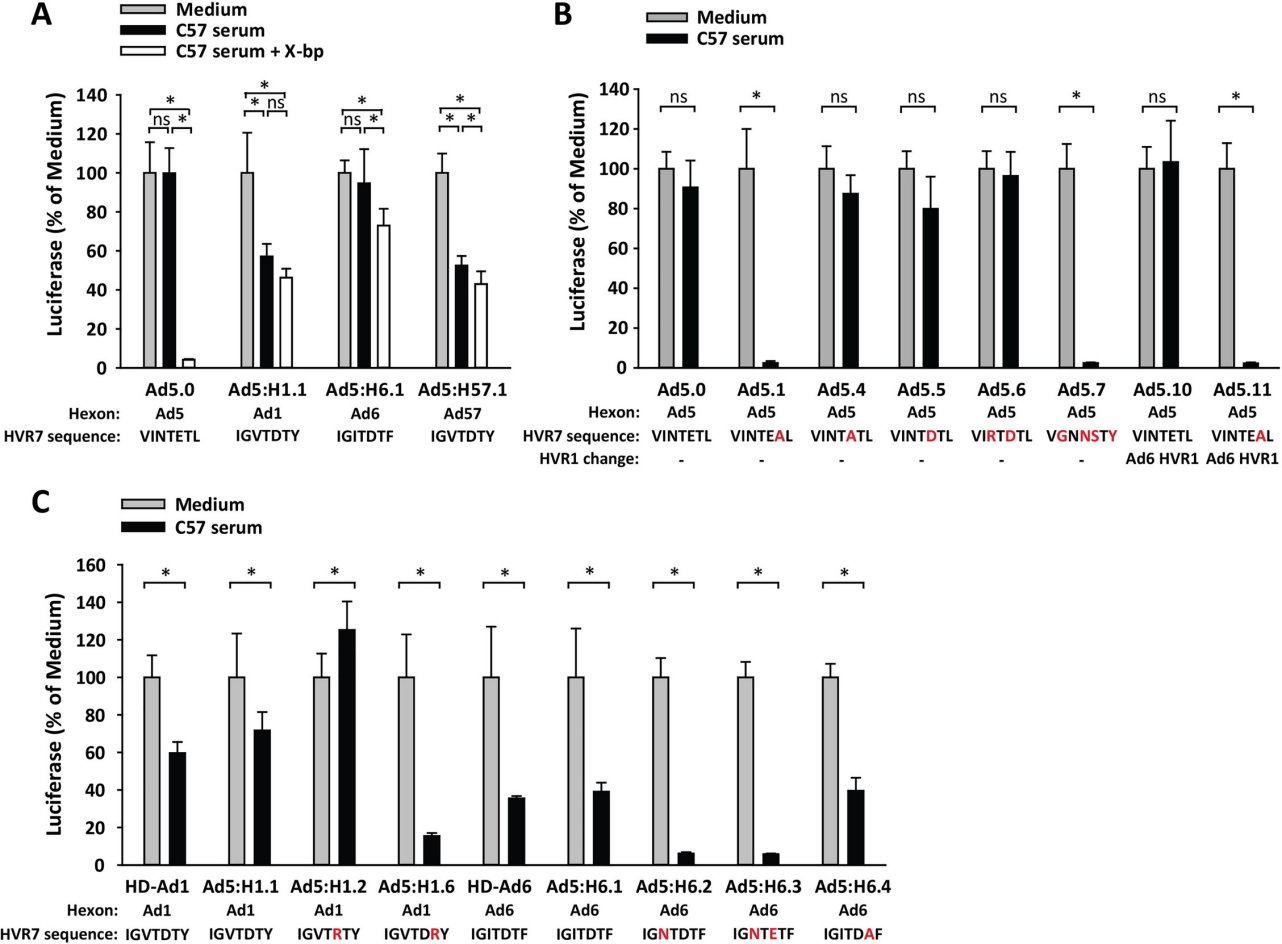
Neutralization of vectors by mouse serum. Ad vectors were incubated with C57BL/6 mouse serum and assessed for ability to transduce 293 cells. X-bp was added to certain samples to block FX. (A) Effect of mouse serum on an Ad5 vector (Ad5.0) as compared to Ad5 vectors with chimeric hexon from Ad1, Ad6 or Ad57. (B) Effect of mouse serum on Ad5.0 and vectors with mutations in Ad5 hexon. (C) Effect of mouse serum on HD-Ad vectors or hexon-chimeric vectors with chimeric hexon from Ad1 or Ad6. Mutated residues in HVR7 are indicated by red letters. *N* = 6 per group. * *P* ≤ 0.05 (ANOVA and Holm-Sidak in panel A; *t*-test in panels B and C).

We next evaluated the ability of mouse serum to neutralize mutated Ad5 vectors and hexon-chimeric vectors. As expected, vectors with Ad5 hexon were neutralized by mouse serum when FX was blocked with X-bp (Fig 7A, vector Ad5.0) or when hexon mutations prevented binding of coagulation factors (Fig 7B, vectors Ad5.1, Ad5.7 and Ad5.11). Ad5 vectors with chimeric hexon from Ad1 and Ad57 were moderately neutralized by mouse serum (Fig 7A). Neutralization of Ad5:6.1 (vector with chimeric Ad6 hexon) was variable among experiments (compare Fig 7A and 7C). Within a single experiment, however, neutralization of HD-Ad6 (which has a complete Ad6 capsid) was similar to neutralization of Ad5:H6.1 (Ad5 capsid with chimeric Ad6 hexon), suggesting that variability in neutralization was due to differences in mouse serum between experiments (Fig 7C). While Ad5 vectors were clearly dependent on FX for protection from neutralization by serum (Fig 7A and 7B), there was little evidence for a strong modifying effect of coagulation factors on neutralization of vectors that have non-Ad5 hexon (Fig 7A and 7C).

### Liver transduction by hexon-chimeric and hexon-mutated Ad vectors

**Fig 8:**
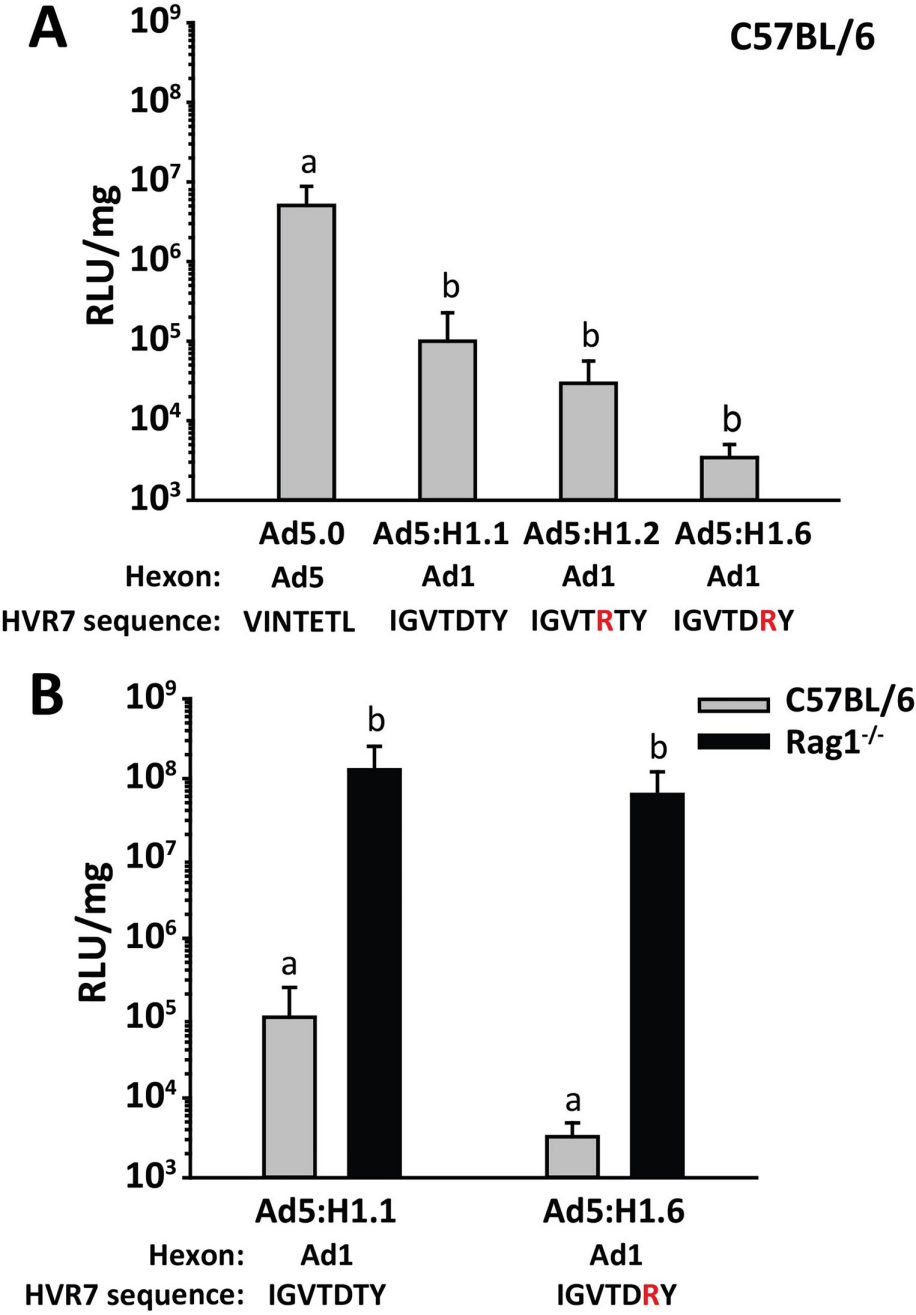
Liver transduction following IV injection of mice with vectors with chimeric hexon from Ad1. Mice were injected IV with 4x10^11^ vp/kg of vector, and liver luciferase expression was measured 48 h later. (A) Liver luciferase expression after IV injection of C57BL/6 mice with Ad5 vector (Ad5.0) or Ad5 vectors with chimeric Ad1 hexon (Ad5:H1.1) or mutated Ad1 hexon (Ad5:H1.2, Ad5:H1.6). Mutated residues in HVR7 are indicated by red letters. (B) Liver luciferase expression after IV injection of *Rag1*^-/-^ mice compared to C57BL/6 mice. *n* = 5 mice per group. Statistical comparison was performed by ANOVA and Holm-Sidak post-hoc tests, and groups that do not share the same letter are significantly different from each other (*P* ≤ 0.05).

To evaluate any role that coagulation factors may play in liver transduction by vectors with Ad1 hexon, we injected C57BL/6 mice IV with Ad5 vectors containing chimeric Ad1 hexon (Ad5:H1.1, Ad5:H1.2 and Ad5:H1.6), and we examined liver luciferase expression. In C57BL/6 mice, we had found that a HD-Ad1 vector (with the entire Ad1 capsid) gave lower liver luciferase expression relative to HD-Ad5 vector (Fig 2B), and similarly we found that Ad5:H1.1 (Ad5 vector with chimeric Ad1 hexon) also showed lower liver transduction relative to Ad5.0 (Fig 8A). This result demonstrates that the reduced ability of Ad1 vector to transduce mouse liver is due to hexon. The mutant chimeric vector Ad5:H1.6, which had extremely low affinity for mouse FX and mouse FII (S2 Fig), was further evaluated in antibody-deficient (*Rag1*^-/-^) mice. Liver luciferase expression by both Ad5:H1.1 and Ad5:H1.6 was greatly enhanced in *Rag1*^-/-^ mice relative to C57BL/6 mice, and both vectors achieved the same level of luciferase expression in *Rag1*^-/-^ mice (Fig 8B). This result demonstrates that vectors with Ad1 hexon are greatly inhibited by natural antibodies. Overall, we conclude that vectors with Ad1 hexon are extremely sensitive to natural antibodies *in vivo*, even when able to bind coagulation factors.

**Fig 9:**
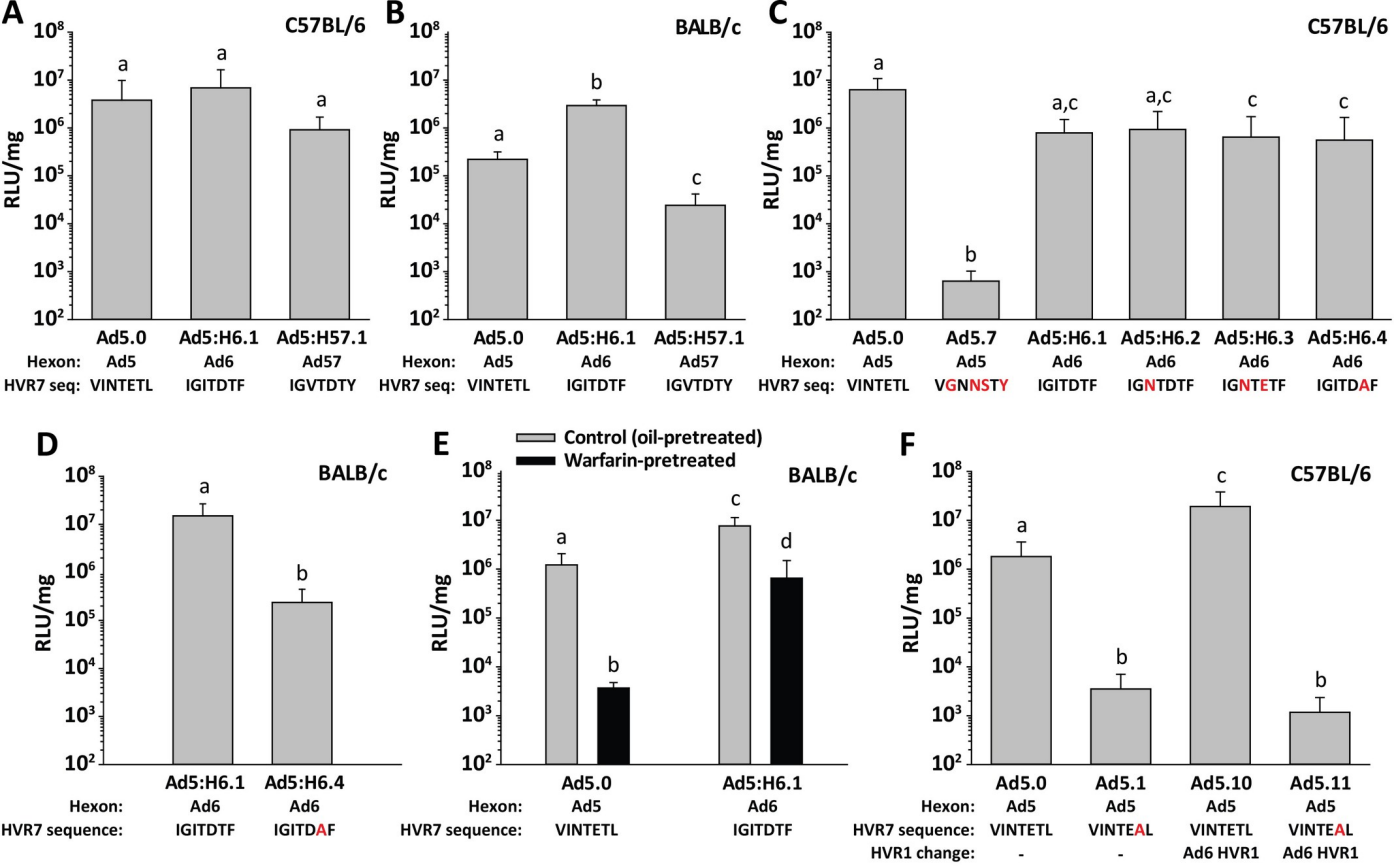
Liver transduction by Ad5 vectors containing chimeric hexon from Ad6 and Ad57. Mice were injected IV with 4x10^11^ vp/kg of vector, and liver luciferase expression was measured 48 h later. Liver luciferase was evaluated after injection of Ad5 vector (Ad5.0) or vectors with chimeric hexon from Ad6 or Ad57 in (A) C57BL/6 mice or (B) BALB/c mice. (C) Liver luciferase after IV injection of C57BL/6 mice with vectors that contain chimeric hexon from Ad6. Mutated residues in HVR7 are indicated by red letters. Liver transduction with Ad5.0 (normal Ad5 vector) and Ad5.7 (Ad5 vector with no ability to bind FX or FII) are included for comparison. (D) Liver luciferase after injection of Ad5:H6.1 or Ad5:H6.4 in BALB/c mice. (E) Effect on liver luciferase activity of depleting coagulation factors with warfarin, prior to injection of Ad5.0 and Ad5:H6.1 in BALB/c mice. (F) Swapping HVR1 from Ad6 into an Ad5 vector and evaluating liver luciferase in C57BL/6 mice. Ad5.0 and Ad5.1 have a normal Ad5 HVR1, while Ad5.10 and Ad5.11 have HVR1 from Ad6. Ad5.0 and Ad5.10 have a normal Ad5 HVR7, while the HVR7 region of Ad5.1 and Ad5.11 have a T425A mutation that abolishes binding of FX and FII. *N* = 4–8 per group. Statistical comparison was performed by ANOVA and Holm-Sidak post-hoc tests, and groups that do not share the same letter are significantly different from each other (*P* ≤ 0.05).

We next evaluated liver transduction with vectors that have chimeric Ad6 or Ad57 hexon. When injected IV into C57BL/6 mice, Ad5:H6.1 and Ad5:H57.1 achieved liver luciferase expression that was similar to Ad5.0 (Fig 9A). In contrast, in BALB/c mice, liver transduction by Ad5:H57.1 was significantly worse than the other two vectors (Fig 9B). We also evaluated the effect of Ad6 hexon mutations that decrease binding of coagulation factors. We found little effect of Ad6 hexon mutations on liver transduction in C57BL/6 mice (Fig 9C), but in BALB/c mice there was a significant reduction in liver transduction with Ad5:H6.4 (Fig 9D), which is a vector that has low affinity for coagulation factors (Fig 6B). We used warfarin pre-treatment to further confirm the effect of coagulation factors on liver transduction. Pre-treating BALB/c mice with warfarin reduced liver transduction by vector with chimeric Ad6 hexon (Ad5:H6.1) by just 12-fold, as compared to warfarin causing a 330-fold reduction in liver transduction for Ad5 vector (Fig 9E). Together with other data on liver transduction by HD-Ad5 and HD-Ad6 (Figs 2 and 3A), these results indicate that liver transduction by vectors with Ad6 hexon exhibits reduced sensitivity to natural antibodies and reduced dependence on coagulation factors, relative to Ad5 vectors.

Swapping Ad6 HVR1 into Ad5 vectors (vectors Ad5.10 and Ad5.11) led to a slight enhancement in liver transduction when hexon was able to bind coagulation factors (Fig 9F, compare Ad5.0 and Ad5.10). However, HVR1 from Ad6 did not improve liver transduction in Ad5 vectors that were ablated for coagulation factor binding (Fig 9F, compare Ad5.1 and Ad5.11). Thus, replacing HVR1 of Ad5 with HVR1 of Ad6 affects neither coagulation factor binding *in vitro* (Figs 6A and S1) nor dependence on coagulation factors for liver transduction *in vivo* (Fig 9F).

## Discussion

The major Ad capsid protein hexon plays a key role in determining the properties of Ad gene therapy vectors. In this study, we found that the closely-related hexons in human Ad species C conferred markedly different properties on Ad vectors, especially after IV injection of mice.

Our observation that species C hexons bind FII is significant because FII is the most abundant coagulation factor in plasma, with a molar concentration that is 8-fold higher than the molar concentration of FX. We found that viruses or vectors with hexons from Ad1, Ad2, Ad6 and Ad57 bound human FII and mouse FII with roughly similar affinity (in the nanomolar range). In contrast, binding of FII to Ad5 was weaker, and in particular mouse FII bound poorly to Ad5, with only micromolar affinity. Binding of mouse FX to all species C hexons was extremely strong (sub-nanomolar affinity), but binding of human FX was more variable among hexons, with only Ad5 showing sub-nanomolar affinity for human FX.

It does not appear that differences in affinity for coagulation factors translate in a straightforward way into different *in vivo* properties of Ad vectors. In particular, we found that vectors based on Ad1, Ad2, Ad6 and Ad57 showed marked differences in liver transduction, differing sensitivity to antibodies, and varying dependence on coagulation factors *in vivo*, in spite of the fact that affinity for mouse FX or mouse FII was similar among these vectors. Nevertheless, the differing affinities of mouse and human coagulation factors for Ad vectors should be kept in mind when interpreting the results of gene therapy studies with Ad vectors in mice and when considering the applicability of mouse studies to human gene therapy.

The snake venom protein X-bp binds to the Gla domain of FX, and can be used to specifically interrogate the role of FX *in vivo* [1,7]. Unfortunately, there is no known reagent that can specifically block the Gla domain of FII. We therefore introduced mutations into the hexon HVR7 region in the hope of specifically ablating binding of FII to hexon. However, hexon mutations that reduced the affinity of FII also simultaneously reduced the affinity of FX. We were able to show that FX and FII bind to the same site on hexon and can even compete with each other in some cases. Overall, however, limitations in experimental tools prevented a clear differentiation of the relative roles of FX and FII in liver transduction by non-Ad5 vectors. While the importance of FX binding is firmly established for Ad5 vectors, further studies are needed to address whether FII binding to Ad5 or non-Ad5 vectors may affect vector biodistribution and toxicity profiles after IV administration.

There has been extensive study of the host proteins and mechanisms that affect systemically-administered Ad5 vectors in mice, including the inhibitory effects of natural antibodies and the protective effect of FX against neutralization by natural antibodies and complement [3,4,7,8,21,30]. One of the major goals of the current study was to determine whether vectors based on non-Ad5 members of Ad species C are affected by these host proteins in a similar manner as Ad5 vectors. Our investigations in mice found that liver transduction by all of the tested species C vectors was negatively affected by natural antibodies and positively affected by coagulation factors, although to different degrees for different vectors. Ad6 was relatively resistant to the negative effects of natural antibodies, showing no major differences in liver transduction between mouse strains that have high concentrations of IgM (BALB/c) or low concentrations of IgM (C57BL/6). In addition, we found that liver transduction by Ad6 in antibody-deficient J_H_D mice was only slightly enhanced relative to liver transduction in BALB/c mice. Liver transduction by the other vectors was more severely suppressed by natural antibodies, most notably for the Ad2 vector.

Regarding the effects of coagulation factors on liver transduction by species C vectors, all of the tested vectors showed some dependence on coagulation factors, but once again vectors with Ad6 hexon were affected only modestly. In fact, vectors with Ad6 hexon showed no requirement for coagulation factors for liver transduction in C57BL/6 mice. BALB/c mice are a much more stringent model for evaluating the robustness of Ad gene therapy vectors due to the higher concentrations of natural IgM antibodies than in C57BL/6 mice [8]. In BALB/c mice, liver transduction by vectors with Ad6 hexon could be partially suppressed by depleting coagulation factors with warfarin or by using a vector with mutant Ad6 hexon that is defective for coagulation factor binding (Ad5:H6.4). In contrast to vectors with Ad6 hexon, liver transduction by vectors with Ad1 hexon was poor due to their high sensitivity to natural antibodies. We found that vectors with chimeric Ad1 hexon were capable of very high liver transduction in antibody-deficient *Rag1*^-/-^ mice, demonstrating that vectors with Ad1 hexon have no inherent difficulty in transducing liver when antibodies are absent.

Although the liver is the major organ sequestering Ad vectors after IV administration, modulating liver-directed vector tropism may lead to unpredictable changes in vector distribution, toxicity profile and transduction of other organs, particularly the spleen [11,53,54]. We only examined liver transduction in the current study, and in future work it will be valuable to explore the potential impact of FII and hexon on biodistribution of vector in other organs, as well as exploring the additional contribution of integrins, which are known to affect biodistribution to the spleen and pro-inflammatory signaling [21,54]. In addition, it is important to point out that the mouse model does not mimic all aspects of human biology. For example, human erythrocytes express CAR, which is a receptor for species C Ad vectors, but mouse erythrocytes do not express CAR [55,56]. Therefore, the mouse model does not take into account that expression of CAR on erythrocytes may influence the biodistribution of species C Ad vectors in humans and other primates.

For Ad5 vectors, our mouse serum model recapitulates the *in vivo* neutralizing effects of natural antibodies and complement, and recapitulates the protective effect of FX in preventing neutralization of Ad5 by natural antibodies and complement [7]. However, when this mouse serum model system was applied to Ad species B and D by Ma *et al*. [3], no neutralization was seen, and FX played no apparent protective role. In the current study, we applied the mouse serum model to all of our vectors. While the results and mechanisms of this model system remain valid for vectors with Ad5 hexon, results with other vectors were less clear-cut and more variable, and the data were much less useful for understanding the *in vivo* behavior of non-Ad5 species C Ad vectors. For example, vectors with Ad6 hexon were partially neutralized in many experiments by serum, even though we found *in vivo* that antibodies had only a limited impact on liver transduction by vectors with unmutated Ad6 hexon. While the neutralization mechanisms that operate in mouse serum appear to involve the same host proteins that influence liver transduction *in vivo*, we conclude that results from the mouse serum model must be interpreted cautiously when using non-Ad5 serotypes, and the mouse serum model cannot be used on its own to predict the ability of non-Ad5 vectors to transduce liver in mice.

In conclusion, we have explored the diversity of gene therapy vectors based on hexons from human Ad species C. We found that liver transduction by all vectors was affected by natural antibodies and coagulation factors, but to markedly different degrees. Ad2 was the most sensitive to natural antibodies, and Ad6 the least sensitive. We also discovered robust binding of all tested viruses and vectors to FII, which is significant because FII is present in the circulation at much higher concentrations than all other coagulation factors. Finally, we caution that coagulation factors from mice and humans sometimes have very different affinities for Ad hexons. This finding indicates a need to carefully evaluate the appropriateness of mouse studies for preclinical studies with novel Ad vectors.

## Materials and methods

### Ethics statement

Procedures using mice were carried out in strict accordance with FDA institutional guidelines and the standards contained in the Guide for the Care and Use of Laboratory Animals (The Guide, 8th Edition) of the National Institutes of Health. Animal protocols were approved by the FDA White Oak Consolidated Animal Care and Use Committee (Protocol Numbers 2003–11 and 2004–16). Animal facilities were accredited by the Association for Assessment and Accreditation of Laboratory Animal Care International.

### Ads and Ad vectors

All E1/E3-deleted Ad5 vectors express luciferase under control of a CMV promoter. Ad5.0 has a wild-type Ad5 capsid and Ad5.7 contains mutations in HVR7 that ablate binding of FX. Construction of these vectors was described previously [44]. Other Ad5 Hexon-chimeric and Ad5 hexon site-directed mutants were constructed in a similar manner using a modified version of the backbone plasmid pAdHM4-CMVL1 [57] in which Ad5 hexon was replaced or mutated by homologous recombination in *E*. *coli*. For hexon-chimeric vectors, we removed Ad5 vector sequences in L3 that encode hexon amino acids 112–593 (which includes all hexon hypervariable regions) and replaced these Ad5 hexon sequences with the corresponding sequences from another Ad species C hexons. Vector plasmids were linearized and transfected into 293 cells. Vectors that were rescuable are shown in Fig 1. A number of additional mutations were made in HVR7 that did not result in rescuable vectors. Vectors were grown on 293 cells, purified by CsCl ultracentrifugation, formulated and characterized as previously described, with concentration in vg/mL determined by absorbance of 260 nm light [7].

All serotypes of HD-Ads were produced according to the methods described in Palmer and Ng [58]. HD-Ad5 was produced using the helper virus AdNG163 [58]. HD-Ad2 was produced using the helper virus Ad2LC8cCARP [59]. HD-Ad1 and HD-Ad6 were produced using the helper viruses Ad1LC8cCEVS-1 and Ad6LC8cCEVS-6, respectively that were generously provided by Carole Evelegh and Frank L. Graham (McMaster University).

Human Ad serotypes Ad1 (VR-1), Ad2 (TR-846), Ad5 (VR-5) and Ad6 (VR-6) were obtained from the American Type Culture Collection (Manassas, VA) and were grown on 293 cells and purified in the same manner as the E1/E3-deleted Ad vectors.

### Mice

Male C57BL/6J and BALB/cJ mice were obtained from Jackson Laboratories. Knockout mice were obtained and bred as previously described [7]. *Rag1*^-/-^ and *C1q*^-/-^ mice are on a C57BL/6 background, and J_H_D mice are on a BALB/c background.

To deplete vitamin K-dependent coagulation factors in selected experiments, mice were subcutaneously dosed with 133 μg of warfarin suspended in peanut oil on days -3 and -1 before injection with vector. Control mice were injected with oil alone.

For IV injection, unanesthetized mice were restrained and injected via the lateral tail vein with 4.0 × 10^11^ vp/kg of vector, and livers were collected as a terminal procedure under anesthesia 48 h later. Mice were anesthetized by intraperitoneal injection of 150 mg per kg body weight ketamine and 30 mg per kg body weight xylazine. Organs were harvested and homogenized in buffer (25 mM Tris-phosphate, pH 7.6; 2 mM EDTA; 10% glycerol; 1% Triton X-100). Luciferase activities were measured using a kit (Promega, E1501). All transgene expression data were normalized to total protein concentration (Bio-Rad, DC Protein Assay Kit, 500–0116).

### Western and neutralization assays using mouse serum

Blood was collected from mice, and serum was processed as previously described [7]. Serum was separated by centrifugation, stored on ice, and used on the same day due to the instability of mouse complement.

Binding of serum proteins was evaluated by quantitative western blot as previously described [7]. Briefly, viruses were mixed with mouse serum, incubated at 37°C for 30 min, and then virions and their bound proteins were separated by ultracentrifugation on a discontinuous Histodenz gradient. The virion fraction was subjected to SDS-PAGE, and western blots were performed using antibodies to detect Ad5, fiber, C3 and FX as previously described [7] or goat anti-mouse IgM (#1021–01; Southern Biotech; 1:1,000 dilution). Blots were scanned and bands were quantified using a LI-COR Odyssey imager. The protein of interest and fiber were detected in separate fluorescent channels. To normalize for differences in protein loading per lane or differences among individual western blots, for each lane we calculated the ratio of the intensity of the band of interest vs. the intensity of the fiber band from the same lane.

For neutralization experiments, 293 cells were plated in 96-well plates at 5 × 10^4^ cells per well, one day before the experiment. In certain experiments, serum was pretreated at 56°C for 30 min to inactivate complement. In certain experiments, 80 μg/ml X-bp was added to block FX. X-bp was chromatographically purified from *Deinagkistrodon acutus* venom and was kindly provided by Takashi Morita [7,60]. Neutralization reaction mixtures were prepared on ice, with Ad vector added as the last component. The final concentration of vector in each 25 μL reaction was 5 × 10^8^ vp/reaction (2 × 10^10^ vp/mL), and the final concentration of serum was 86%. Negative control samples (baseline transduction) consisted of vector at 2 × 10^10^ vp/mL in serum-free DMEM supplemented with 2% globulin-free BSA (Sigma-Aldrich). Neutralization reactions were incubated at 37°C for 30 min and then placed on ice. Neutralization mixtures were diluted 2,000-fold in serum-free DMEM. 293 cells were rinsed once with serum-free DMEM, and then 100 μL of diluted neutralization mixtures (containing 1 × 10^6^ vp) was added to each well and incubated at 37°C and 5% CO_2_ for 2 h. Following this incubation, the inoculum was replaced with medium containing 10% FBS. After approximately 18 h, cells were rinsed with PBS once and then lysed by incubation in 150 μL of lysis buffer (25 mM Tris-phosphate, pH 7.6; 2 mM EDTA; 10% glycerol; 10% Triton X-100) at 4°C with 600 rpm orbital shaking for 30 min. Following lysis, plates were spun for 5 min at 1,500 rpm at 4°C to pellet cellular debris. 20 μL of lysate for each reaction was then transferred to a white-walled 96-well plate and injected with 100 μL of Luciferase Assay System Reagent (Promega, E1501) using the manufacturer’s protocol for a plate-reading luminometer (Glomax, Promega). Protein concentrations of lysates were determined by diluting 15 μL of lysate in 135 μL of water and then performing a Micro BCA Protein Assay (ThermoFisher, 23235) according the manufacturer’s instructions.

### Effect of coagulation factors on cell transduction

SKOV3 cells (HTB-77, American Type Culture Collection) were seeded in 96-well plates at 5 × 10^4^ cells per well, one day prior to the experiment. Purified human FX (HCX-0050) and human FII (HCP-0010) were purchased from Haematologic Technologies, Inc. HD-Ad vectors (2 × 10^8^ vp/mL) were incubated for 30 min on ice with 10 μg/ml of FX and/or 100 μg/ml of FII in PBS containing calcium and magnesium. SKOV3 cells were rinsed once with PBS, and then 100 μL of each vector mixture (containing 2 × 10^7^ vp) was added to triplicate wells. After incubation at 37°C for 2 h, vector was removed and replaced with culture medium. After approximately 18 h, cells were rinsed with PBS once and cell lysate was assayed for luciferase activity as described above.

### Affinity measurement using SPR

Plasma-derived coagulation factors were purchased from Haematologic Technologies, Inc.: human FX (HCX-0050), mouse FX (MCX-5050), bovine FX (BCX-1050), human FII (HCP-0010), mouse FII (MCP-5010) and bovine FII (BCP-1010). Concentrations of coagulation factors were determined by measurement of light absorbance at 280 nm. Because we found that FX preparations contain small amounts of FXa protease activity that interferes with SPR experiments for certain Ad types, we pretreated FX with a specific protease inhibitor before SPR experiments. GGACK (EGRCK) is a competitive inhibitor that covalently attaches to the active site of FXa [61]. GGACK (Haematologic Technologies, Inc.) was added to FX at a final concentration of 125 μM and incubated on ice for 30 min. Before use in SPR experiments, all coagulation factors and Ad vectors were dialyzed at 4°C with 10 mM HEPES, 150 mM NaCl, 1 mM CaCl_2_, 0.5 mM MgCl_2_, pH 7.4.

SPR experiments were performed on a Biacore T200 system (GE Healthcare). To perform SPR, approximately 300 to 5,000 reference units (RU) of Ad was immobilized on a CM5 Biacore sensor chip. Running buffer was 10 mM HEPES, 150 mM NaCl, 1 mM CaCl_2_, 0.5 mM MgCl_2_, 0.1% (wt/vol) bovine serum albumin and 0.05% (vol/vol) polysorbate 20, pH 7.4. Coagulation factors were diluted in running buffer to four concentrations (one of which was run in duplicate). The flow rate was 50 μL/min, and in some experiments an extended dissociation time was used for one run to aid in obtaining an accurate off-rate. Because coagulation factor binding depends on divalent cations, sensors were regenerated for 2 min with running buffer in which the CaCl_2_ and MgCl_2_ were replaced with 3 mM EDTA. Regeneration was followed by equilibration for 1 min in running buffer. Data were acquired at 1 Hz and globally fit to a 1:1 kinetic interaction model using Biacore T200 Evaluation Software 3.0, with double referencing.

### Statistics

Statistical analyses were performed using SigmaPlot (Systat Software). Data were log-transformed to equalize variances when necessary, and then evaluated using either one-way ANOVA or unpaired *t*-test, as indicated in the figure legends. For ANOVA, post-hoc pairwise comparisons were performed with the Holm-Sidak test.

## Supporting information

S1 Fig Binding of FX and FII to Ad5 vectors with mutations in hexon HVR7. SPR plots supporting Fig 6A, showing FX and FII binding to an Ad5 vector with wild-type Ad5 capsid (Ad5.0) and to Ad5 vectors with various mutations in hexon. Mutated residues in HVR7 are indicated by red letters. Ad5.10 and Ad5.11 contain the Ad6 HVR1 region. Kinetic binding affinities are shown in nM. NB: No binding.
(PDF)

S2 Fig Binding of FX and FII to Ad5 vectors with chimeric hexon from Ad1, Ad6 or Ad57. SPR plots supporting Fig 6B, showing FX and FII binding to hexon-chimeric vectors. Mutated residues in HVR7 are indicated by red letters. Kinetic binding affinities are shown in nM. NB: No binding. Graphs marked “cannot be determined” showed evidence of very low-affinity binding, but the data could not be fit using a 1:1 binding model due to complex binding curves and/or off rates that were too rapid to measure accurately.
(PDF)

S1 Data Raw data from this study. (XLSX)
