# Supplementary figures and images for: Binding of adenovirus species C hexon to prothrombin and the influence of hexon on vector properties in vitro and in vivo
Source: PLoS Pathog. 2022 Sep 26;18(9):e1010859. doi: 10.1371/journal.ppat.1010859 (PMC9536601; doi:10.1371/journal.ppat.1010859)

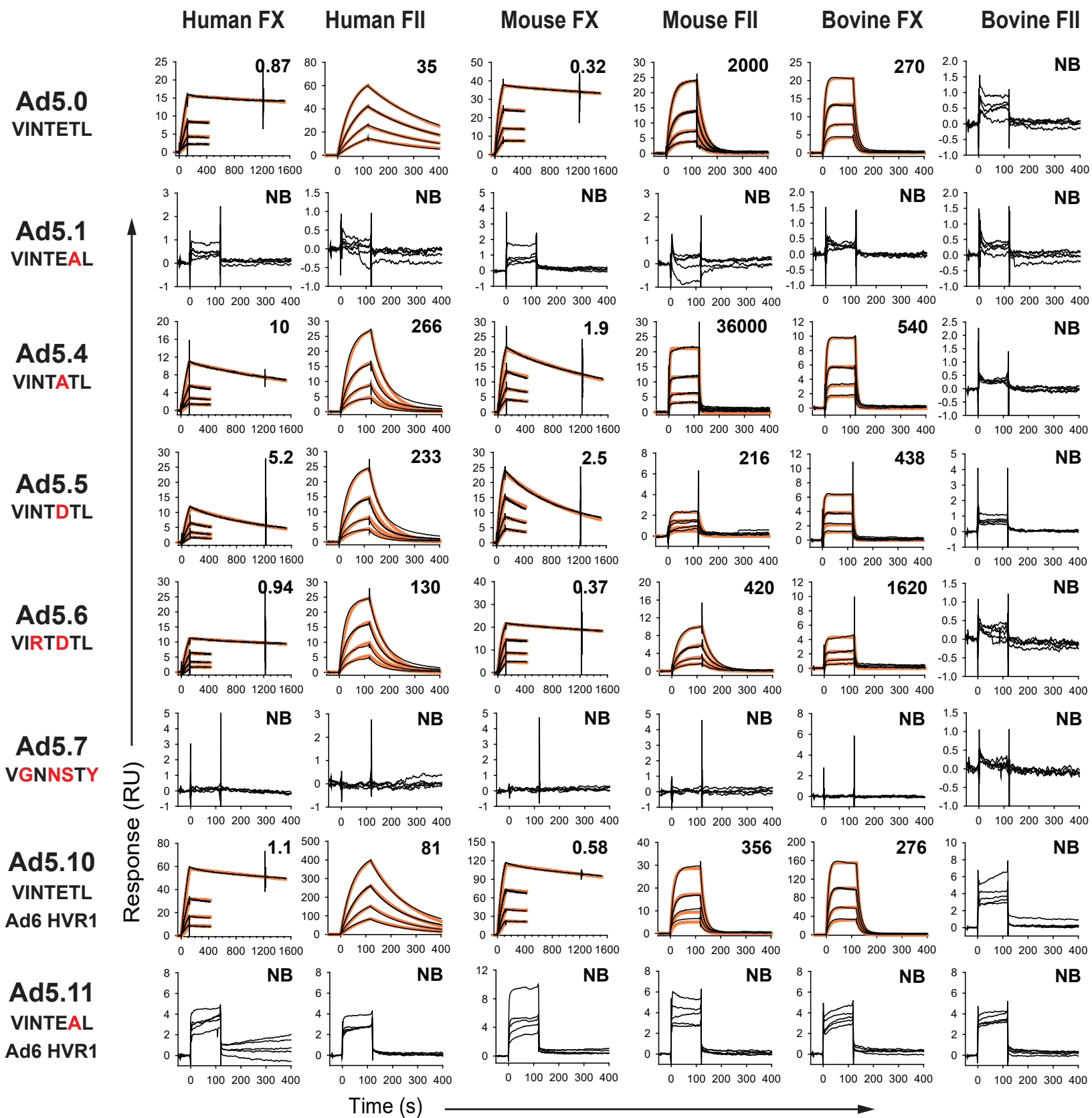

Supplement: S1 Fig — SPR plots supporting Fig 6A, showing FX and FII binding to an Ad5 vector with wild-type Ad5 capsid (Ad5.0) and to Ad5 vectors with various mutations in hexon. Mutated residues in HVR7 are indicated by red letters. Ad5.10 and Ad5.11 contain the Ad6 HVR1 region. Kinetic binding affinities are shown in nM. NB: No binding. (PDF) [file ppat.1010859.s001.pdf]

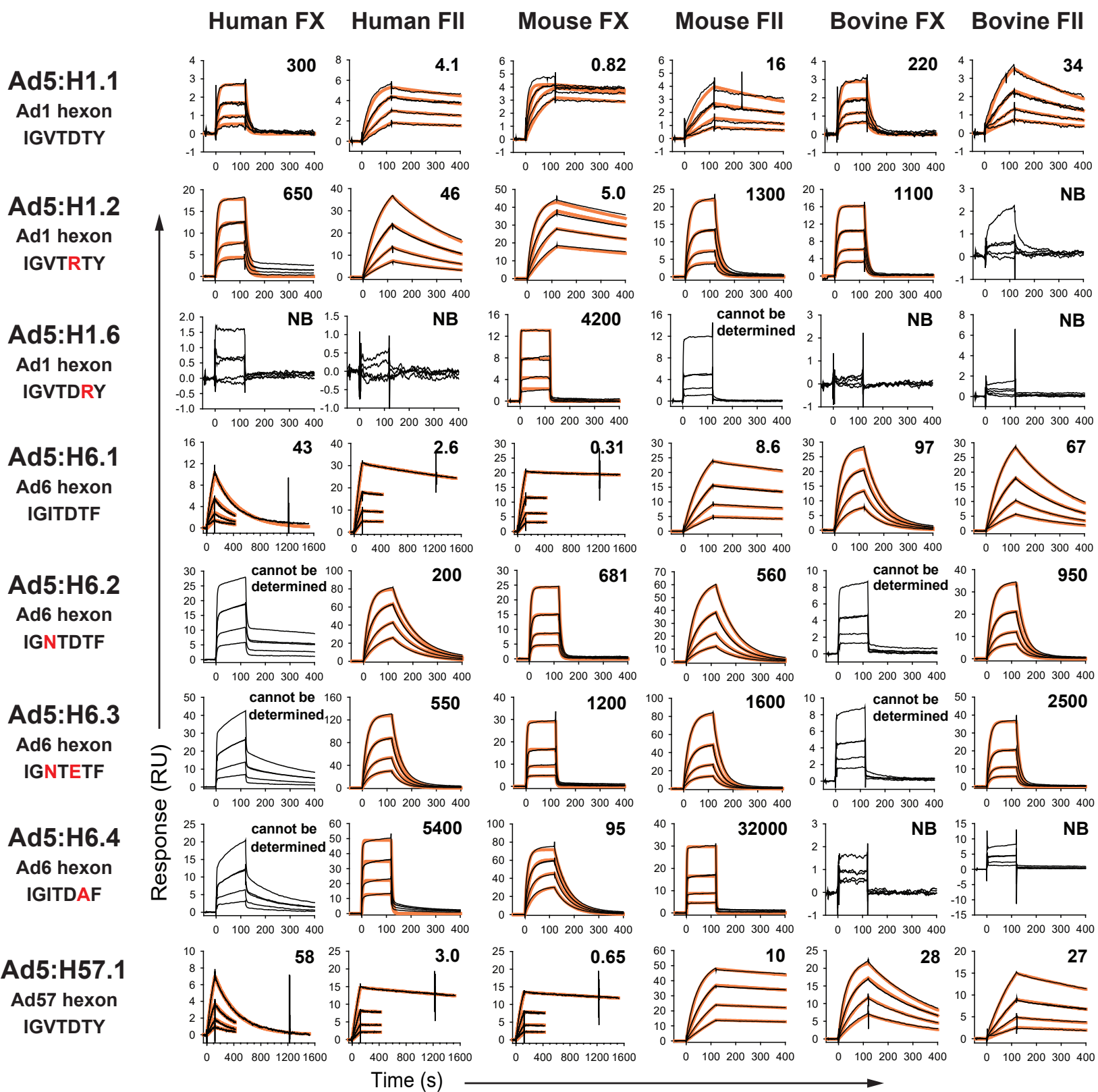

Supplement: S2 Fig — SPR plots supporting Fig 6B, showing FX and FII binding to hexon-chimeric vectors. Mutated residues in HVR7 are indicated by red letters. Kinetic binding affinities are shown in nM. NB: No binding. Graphs marked “cannot be determined” showed evidence of very low-affinity binding, but the data could not be fit using a 1:1 binding model due to complex binding curves and/or off rates that were too rapid to measure accurately. (PDF) [file ppat.1010859.s002.pdf]
